# Long non-coding RNAs in intracerebral hemorrhage

**DOI:** 10.3389/fnmol.2023.1119275

**Published:** 2023-06-12

**Authors:** Chenyu Zhang, Ying Zhang, Qi Wang, Zhenwei Fang, Xinyi Xu, Mengnan Zhao, Ting Xu

**Affiliations:** ^1^Department of Pharmacy, West China Hospital, Sichuan University, Chengdu, China; ^2^West China School of Pharmacy, Sichuan University, Chengdu, China

**Keywords:** intracerebral hemorrhage, long non-coding RNA, pathology, therapeutic target, hemorrhagic stroke

## Abstract

Intracerebral hemorrhage (ICH), a subtype of stroke, can lead to long-term disability and is one of the leading causes of death. Unfortunately, the effectiveness of pharmacological therapy for ICH is still uncertain. Long non-coding RNA (lncRNA) was defined as an RNA molecule that consists of more than 200 nt without translational activity. As a vital class of diverse molecules, lncRNAs are involved in developmental and pathological processes and have been attractive for decades. LncRNAs have also become potential targets for therapies, as they were massively identified and profiled. In particular, emerging evidence has revealed the critical role of lncRNAs in ICH while attempts were made to treat ICH via regulating lncRNAs. But the latest evidence remains to be summarized. Thus, in this review, we will summarize the recent advances in lncRNA in ICH, highlighting the regulatory role of lncRNAs and their potential as therapeutic targets.

## 1. Introduction

Intracerebral hemorrhage (ICH), a subtype of stroke, refers to the spontaneous rupture of injured small arteries or arterioles, leading to blood accumulation in cerebral parenchymal (Steiner et al., 2014; Gross et al., 2019). Although ICH counts for a smaller portion of all types of strokes (9–27%; Feigin et al., 2009; Sacco et al., 2009; Steiner et al., 2014), the global burden of ICH is higher than that of ischemic stroke (Krishnamurthi et al., 2013). A meta-analysis of nine studies showed that the mortality rate could be as high as 35.3% 3 months after ICH onset (Pinho et al., 2019).

Acute interventions for ICH, including medical therapies and minimally invasive surgery are likely to improve acute outcomes. Besides, sustained blood pressure control and optimized antithrombotic therapy are regarded as essential preventive strategies for improving longer-term outcomes in ICH (Hostettler et al., 2019). A better understanding of how the pathological mechanisms drive neurological injury in individuals is urgently required in order to develop therapies for acute and secondary progressive stages of ICH. Research on long non-coding RNA (lncRNA) has been attractive for decades. Up to now, a variety of lncRNAs have been proven to be involved in cerebral development and diseases, such as degeneration diseases of the central nerve system (Scheele et al., 2007; Chiba et al., 2009; Lagier-Tourenne et al., 2012; Ciarlo et al., 2013) and stroke (both ischemic and hemorrhagic stroke; Zhang and Wang, 2019). With the gain- and loss-of function methods, the functions of lncRNAs in ICH were further studied.

In this regard, it is crucial to reveal the current knowledge about the roles of lncRNAs in ICH. Therefore, we will briefly review the involvement of lncRNAs in the ICH-caused damages and their potential to be therapeutic targets and biomarkers will be discussed. The searching strategies used in this article are summarized in Supplementary material.

## 2. The pathogenesis of acute and secondary insults after ICH

Quickly after vessel rupture, blood accumulates in the cerebral parenchymal, causing intracranial pressure elevation, perilesional edema, and structural damage, which may lead to brain hernia and can be fatal (Wilkinson et al., 2018). Mostly, bleeding stops shortly after ICH, but 14–22% of ICH patients may experience hematoma expansion in 6–24 h after ICH onset, which causes more severe structural damage and neurological deterioration, and leads to worse outcomes (Kazui et al., 1996, 1997; Fujii et al., 1998).

After the primary mechanical injury, complicated pathological responses will be triggered. The over-activated microglia release several cytokines and contributes to inflammation, blood brain barrier (BBB) breakdown, and edema in turn (Taylor and Sansing, 2013). Components of the complement system can pass through the damaged BBB and form membrane attack complex, enhancing the BBB injury and neurological damage (Xi et al., 2006). Heme and iron will be released during the erythrocyte elimination process (Wagner et al., 2003). The debris of blood enhances the production of free radicals and contributes to neurological injury, inflammation, BBB damage, and edema (Garton et al., 2016; Yang et al., 2016). Also, excitotoxicity molecules, such as glutamate was shown to anticipate in brain injury after intracerebral hemorrhage (Sharp et al., 2008). Although lots of efforts were made, the full picture of the ICH damage mechanisms, especially of the secondary injury, remains to be further explored.

After the acute stage, microglia polarize toward M2-like microglial, contributing to hematoma clearance (Lan et al., 2017). The hematoma breaks down with the invasion of macrophages and microvessel formation. The hemosiderin-stained scar, a cavity containing blood surrounded by fibrous tissue, and eventually gliosis will ultimately form (Fewel et al., 2003). And neuronal plasticity allows the brain to cope better with the indirect effects of brain damage resulting from ICH (Keep et al., 2012).

## 3. Insights into biological roles of lncRNAs in ICH

### 3.1. Introduction to lncRNA

Long non-coding RNA is commonly defined as an RNA molecule that consists of more than 200 nt without translational activity (Ponting et al., 2009; Nagano and Fraser, 2011). There are at least 170,000 lncRNAs found in humans and more than 130,000 lncRNAs identified in mice (Zhao et al., 2021). In accordance with the relationships between lncRNAs and their regulated genes, lncRNAs were classified as sense lncRNA, antisense lncRNA, intronic lncRNA, intergenic lncRNA, enhancer lncRNA, and circular lncRNA (Uchida and Dimmeler, 2015).

Long non-coding RNAs were long regarded as transcriptional garbage or transcriptional noise. Studies on lncRNA H19 and lncRNA Xist have initially revealed the biological functions of lncRNAs (Brannan et al., 1990; Wilusz et al., 2009; Gendrel and Heard, 2014). Briefly, lncRNAs can interact with proteins, DNA and RNA transcripts to control alternative splicing, chromosome remodeling, nuclear import and mRNA decay, and lncRNAs participate in almost every aspect of gene expression programs (Schmitz et al., 2010; Grote et al., 2013; Khorkova et al., 2015; Isoda et al., 2017).

In recent years, lncRNAs have been characterized are implicated in diverse diseases, including cardiovascular diseases (Uchida and Dimmeler, 2015), neurodegeneration diseases, ischemic stroke, and traumatic brain injury (Riva et al., 2016; Zhang and Wang, 2019; Ren et al., 2020).

### 3.2. LncRNAs play versatile roles in pathological processes underlying ICH

Several studies using high-throughput RNA-seq technique were conducted on mice or rat ICH model, and human samples. In a collagenase-induced mice ICH model, 31 lncRNAs were found to differentially express 24 h after modeling (Hanjin et al., 2018). And another study carried out on a similar model showed 625 dysregulated lncRNAs 21 days after ICH onset (Cao et al., 2020). A similar dynamic change of lncRNAs was observed in rats. Kim et al. (2019) found there were 83, 289, and 401 lncRNAs significantly upregulated and 52, 459, and 786 lncRNAs significantly downregulated 1, 3, and 7 days after collagenase-induced ICH modeling, respectively. These studies revealed the extensive involvement and the dynamic changes of lncRNAs in ICH.

RNA-sequencing data from GSE24265 (containing four human patients’ RNA-seq data) were re-analyzed by Liu et al. (2021) and Yang et al. (2022). Liu et al. (2021) predicted that nine lncRNAs were associated with MAPK1 and may contribute to the progression of ferroptosis after ICH. Yang et al. (2022) found six hub lncRNAs and constructed the potential ceRNA network. In the peripheral blood of ICH patients, 211 lncRNAs dysregulated and were classified into16 lncRNA modules by weighted gene co-expression network analysis and some immune-related lncRNAs were also identified by using ceRNA network (Hao et al., 2022).

These studies supported the advantages of high-throughput techniques in the discovery, functional prediction, and key regulator identification of lncRNAs. However, the exact functions of dysregulated lncRNAs detected by RNA-seq remain to be explored and validated. And the different characteristics of lncRNA dysregulation between species, modeling methods and the less conserved characters of lncRNAs (Sharma and Carninci, 2020) require further study and more careful interpretation from preclinical study results.

Long non-coding RNA H19 (also known as H19 imprinted maternally expressed transcript) was one of the earliest identified lncRNAs (Brannan et al., 1990). H19 was observed to be one of the most stable lncRNAs in the gray matter of human brain and was extensively studied in the development and diseases of the central nerve system, including ischemic stroke, glioma, pituitary adenoma, neuroblastoma, degeneration, and trauma (Zhong et al., 2021). Recently, the role of H19 in ICH was extensively studied. In Kim’s study, lncRNA H19 was the most upregulated lncRNA from day 1 through day 7 after ICH both in the ICH model induced by collagenase or autologous blood (Kim et al., 2019). Further bioinformatic analysis predicted that H19 was associated with type I interferon signaling pathway. Following this study, Chen and colleagues further studied the roles of H19 in ICH. After confirming the high expression level of H19 in the ICH cell model, Chen B. et al. (2021) demonstrated that H19 targeted miR-106b-5p and thus regulated ACSL4, enhancing ferroptosis in brain microvascular endothelial cells (BMVECs) under oxygen and glucose deprivation hemin-treated (OGD/H-treated) condition, as validated by RNA pull-down and luciferase reporter gene assays. In ICH rat model induced by type IV collagenase, NF-κBp65 and IKKβ expression were significantly lower and IκBα was significantly higher in the sh-H19 group when compared with ICH model group, indicating that H19 may be associated with NF-κB pathway. Mao et al. (2022) also found that elevated level of H19 expression was associated with the levels of TNF-α, IL-6, IL-1β, ROS, and MDA, showing that H19 was also associated with inflammation and oxidative stress. Also, H19 was found to be associated with the risk of symptomatic ICH in ischemic stroke patients after recombinant tissue plasminogen activator treatment (Han et al., 2022). Collectively, H19 could interact with miRNA and was demonstrated to be associated with NF-κB pathway, inflammation, free radical production and cell death after ICH.

The lncRNA FOXF1 adjacent non-coding developmental regulatory RNA (FENDRR) increased in C57BL/6 mice with hypertensive ICH (Dong et al., 2018) and was demonstrated to target miR-126 by RNA immunoprecipitation and RNA pull-down. Via targeting miR-126, FENDRR regulated VEGFA and thus contributed to the apoptosis of human brain microvascular endothelial cells. Importantly, it was demonstrated that VEGFA was important in the activation of phosphatidylinositol 3-kinase (PI3K)/protein kinase B (AKT; Ruan and Kazlauskas, 2012).

Xie et al. found that MEG3 elevated in collagenase-induced ICH rat brain tissues. The interaction of MEG3 and miR-181b was validated by Starbase analysis and dual-luciferase reporter assay. And they proved the interaction of MEG3 and miR-181b was associated with the activity of PI3K/AKT pathway. In this study, upregulation of MEG3 was associated with the release of inflammatory cytokines and oxidative stress, contributing to brain edema, neuronal apoptosis, and increased caspase3 activity, which can be reversed by miR-181b inhibition (Xie B. et al., 2021). More importantly, the relationship of MEG3 and miR-181b was confirmed in patients with severe ICH. The upregulation of MEG3 and the downregulation of miR-181b were also observed in serum from ICH patients (Wang H. et al., 2022).

Similarly, lncRNA FGD5-AS1 was also found to regulate PI3K/AKT pathway in collagenase-induced C57BL/6 mice ICH model, although via targeting miR6838-5p/VEGFA axis, which was validated by luciferase reporter gene and pull-down assays. The interaction between FGD5-AS1 and miR6836-5p led to inhibited cell proliferation, increased pro-inflammatory factors, and injured BBB. The proinflammatory effects of FGD5-AS1 were also verified in BMVECs. This study also detected the upregulation of FGD5-AS1 in the serum of ICH patients (Jiang et al., 2022).

GAS5, an extensively studied lncRNA in ischemic stroke, also played important roles in ICH. Wang and colleagues found that FoxO1 could enhance the expression of GAS5 by binding to its promoter. GAS5 bond miR-378a-5p and upregulated the expression of Hspa5, which caused a significant elevation of pro-inflammatory factors, brain edema and neurological injury (Wang B. et al., 2022). And FoxO1 was demonstrated to be a major PI3K-AKT downstream effector (Xing et al., 2018).

Chen and colleagues established the ICH model in mice by collagenase injection and in the cellular inflammation model by treating microglia with lipopolysaccharide. After sequencing RNAs obtained from mice, they found the expression of NONMMUT023599.2 was significantly upregulated. Thus, NONMMUT023599.2 knocking down was conducted in both ICH mice and cellular inflammation model, which strongly downregulated the expression of TRIF, p65 phosphorylation and the secretion of TNF-α and IL-1β, indicating the regulatory potential of NONMMUT023599.2 in NF-κB pathway. The elevation of miR-709, which was previously predicted to be the target of NONMMUT023599.2, was also observed. Authors concluded that elevated levels of NONMMUT023599.2 targeted miR-709, regulated NF-κB pathway, enhanced inflammatory cytokine secretion (such as TNF-α and IL-1β) and thus worsened the outcome after ICH in mice (Chen et al., 2020).

Nuclear factor-k-gene binding interacting lncRNA (NKILA) was first identified in breast cancer. NKILA binds to NF-κB/IκB, masks the phosphorylation motifs of IκB, and thereby inhibits IKK-induced IκB phosphorylation and NF-κB activation. The interaction of NKILA and NF-κB pathway signaling molecules prevented the over-activation of NF-κB pathway (Liu et al., 2015). Zhang et al. also proved that NKILA got involved in the pathological changes after ICH on a collagenase-induced rat model (Jia et al., 2018). NKILA was downregulated in the collagenase-induced rat ICH model. Interestingly, further inhibition of NKILA by siRNA activated NF-κB pathway, reduced endoplasmic reticulum stress, neuron autophagy and neurological deficits, but exacerbated brain edema, neuronal cell apoptosis, BBB breakdown, and promoted inflammatory cytokines release.

The c-Jun N-terminal kinase (JNK) signaling pathway also regulates both physiological and pathological processes, such as neurodegenerative diseases, and inflammatory diseases. Dual specificity phosphatases (DUSPs) were found to regulate the JNK pathway by dephosphorylating their substrates, e.g., DUSP6 binds to JNK2/3 and thus inhibits JNK phosphorylation (Ha et al., 2019). LncRNA TCONS_00145741 could disrupt the interaction between DUSP6 and JNK and stabilize JNK phosphorylation, which suppressed M2 differentiation of microglia after ICH (Wu et al., 2021).

Other studies also pointed out the important roles of some lncRNAs in ICH, although, lack specific targets. Zhang et al. found that SNHG3 expression was significantly induced both in OGD/H-treated BMVECs and in the collagenase-induced rat ICH model. The overexpression of SNHG3 upregulated TWEAK and its receptor Fn14, activating the downstream neuroinflammatory pathway STAT3 and enhancing the expression of matrix metallopeptidase 2/9, causing dysfunction of cerebral microvascular cells after ICH (Zhang et al., 2019). In the autologous blood injection ICH mice model, BLNC1 was upregulated in perihematomal edema, hematoma and microvessel. The elevation of BLNC1 enhanced the apoptosis of BMVECs, potentially by activating PPAR-γ/SIRT6/FoxO3 pathway (Xie L. et al., 2021).

**Figure 1:**
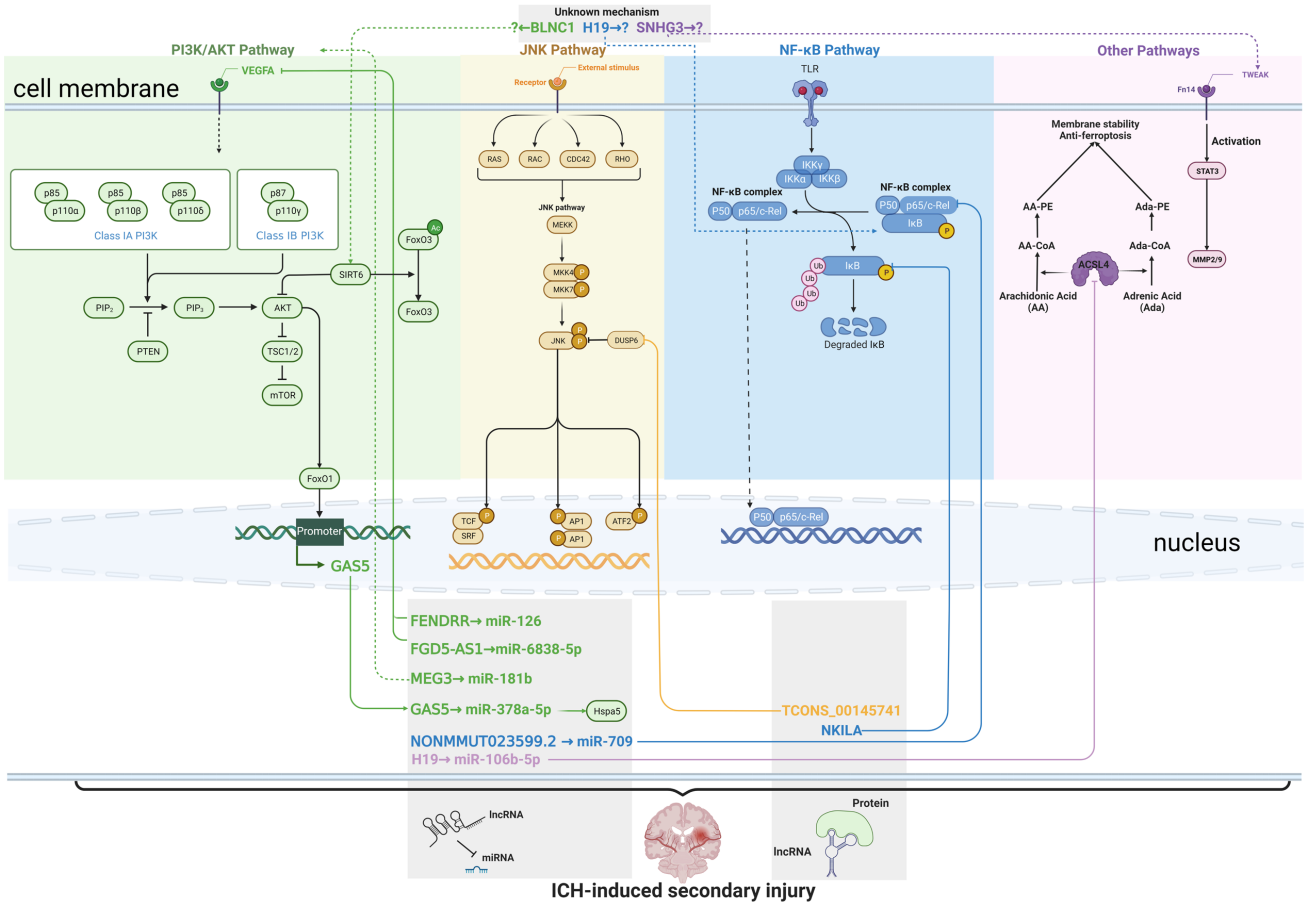
Current knowledge about long non-coding RNAs (lncRNAs) in intracerebral hemorrhage (ICH)-induced secondary injury. Solid lines indicate direct interaction, while dashed lines indicate unknown or indirect regulation. The lncRNAs are marked in green, orange, blue, and purple to indicate their regulation of (or modulation by) the PI3L/AKT pathway, JNK pathway, NF-κB pathway, and other pathways, respectively. The lncRNAs in the same gray box share the similar action mechanisms, such as interacting with miRNAs or proteins. FENDRR, FGD5-AS1, GAS5, NONMMUT023599.2, and H19 were reported to interact with miRNAs, while TCONS_00145741 and NKILA were reported to interact with proteins that contributed to pathological changes after the onset of ICH.

The current knowledge about lncRNAs in ICH-induced secondary injury was summarized in Figure 1. The studies included in this section and the locations of the corresponding lncRNAs are summarized in Table 1. Although many of the lncRNAs were studied in mice or rats, most of them were named in their original articles with capital letters, so we named them with capital letters.

**Table 1:** Summary of studies concerning lncRNAs in ICH.

lncRNA	Location^*^	Target	Subject	Modeling method/Study design	Main finding	Author and publishing year
H19	11p15.5	Unknown	SD rat	cICH and bICH	H19 was the most upregulated lncRNA and was associated with type I interferon signaling pathway.	Kim et al. (2019)
		miR-106b-5p	BMVEC	OGD/H-treated	H19 targeted miR-106b-5p and thus regulated ACSL4, contributing to ferroptosis.	Chen Y. et al. (2021)
		Unknown	SD rat	cICH	H19 may be associated with NF-κB pathway.	Mao et al. (2022)
		Unknown	Human	Cohort study	H19 was associated with the risk of symptomatic ICH in ischemic stroke patients after recombinant tissue plasminogen activator treatment.	Han et al. (2022)
FENDRR	16q24.1	miR-126	C57BL/6 mice	hICH	FENDRR contributed to the apoptosis of BMVEC.	Dong et al. (2018)
MEG3	14q32.2	miR-181b	SD rat	cICH	MEG3 was associated with the release of inflammatory cytokines and oxidative stress.	Xie B. et al. (2021)
		Unknown	Human	Cross-sectional study	The upregulation of MEG3 and the downregulation of miR-181b were also observed in serum from ICH patients.	Wang H. et al. (2022)
FGD5-AS1	3p25.1	miR6838-5p	1. C57BL/6 mice	1. cICH	1. The interaction between FGD5-AS1 and miR6836-5p led to inhibited cell proliferation, increased pro-inflammatory factors and injured BBB. 2. The upregulation of FGD5-AS1 was observed in the serum of ICH patients.	Jiang et al. (2022)
			2. human	2. cross-sectional study		
GAS5	1q25.1	miR-378a-5p	C57BL/6 mice	bICH	GAS5 contributed to the significant elevation of pro-inflammatory factors, brain edema and neurological injury.	Wang B. et al. (2022)
NONMMUT023599.2	15qD1	miR-709	C57BL/6 mice	cICH	NONMMUT023599.2 regulated NF-κB pathway.	Chen et al. (2020)
NKILA	20q13.31	IκB	SD rat	cICH	Inhibition of NKILA after ICH activated NF-κB pathway, reduced neurological deficits, although exacerbated brain edema and BBB breakdown.	Jia et al. (2018)
TCONS_00145741	Not reported	DUSP6 and JNK	C57BL/6 mice	bICH	TCONS_00145741 stabilized JNK phosphorylation and suppressed M2 differentiation of microglia after ICH.	Wu et al. (2021)
SNHG3	1p35.3	Unknown	SD rat	cICH	SNHG3 upregulated TWEAK and its receptor Fn14, which were associated with neuroinflammatory pathway STAT3.	Zhang et al. (2019)
BLNC1	Not reported	Unknown	C57BL/6 mice	bICH	After ICH, Blnc1 activated PPAR-γ/SIRT6/FoxO3 pathway and enhanced the apoptosis of BMVEC.	Xie L. et al. (2021)

BMVEC, brain microvascular endothelial cells; bICH, autologous blood-induced intracerebral hemorrhage; cICH, collagenase-induced intracerebral hemorrhage; hICH, hypertensive hypertension; OGD/H-treated, oxygen and glucose deprivation hemin-treated; and SD rat, sprague dawley rat.

* In human chromosome.

### 3.3. LncRNAs are implicated as diagnose markers and therapeutic targets for ICH

The pathological process of ICH is complicated, thus if the treatment targets versatile regulators, the outcomes of ICH might be improved. Therefore, lncRNAs were capable to be the targets. This assumption was proven right, as knockdown of H19 in ICH rats significantly reduced inflammatory response, oxidative injury, and improved neurological function (Mao et al., 2022). Several drugs were proved to alleviate ICH-induced injury via regulating lncRNAs. After treating ICH rats with Buyang Huanwu decoction, the traditional Chinese formula, Cui et al. found 18 lncRNAs related to ICH were regulated (Cui et al., 2018). Paeonol, a natural product derived from *Paeonia Suffruticosa Andr*., was found to inhibit ferroptosis by regulating lncRNA HOTAIR/UPFI/ACSL4 axis and thus inhibit the progression of ICH (Jin et al., 2021). These two studies showed that lncRNAs involved in ICH could be regulated by drugs, and the regulation of lncRNAs ameliorated ICH-induced damage. More importantly, the results of Cui’s study indicated that numerous lncRNAs can be regulated simultaneously and contributed to better outcomes.

Although we now know that lncRNAs may control the secondary injury in ICH, the specific targets were not extensively studied. lncRNAs can interact with several types of macromolecules, but only limited miRNAs and signaling molecules as targets were further analyzed and validated, while some studies even lacked the validation of targets and numerous lncRNAs were not studied. Lacking specific targets impede drug R&D, even when the transcriptomics data provide hints (Dugger et al., 2018). Identifying the targets and validating the exact functions of lncRNAs in ICH remain attractive and important for drug R&D. Also, efforts were made to use CRISPR/Cas9, siRNAs, etc. to treat diseases by targeting lncRNAs. Viral, liposomes and exosomes might be used to deliver lncRNAs to peri-hematoma tissue (Chen Y. et al., 2021). However, stable targeting delivery systems are still lacking. Although most pharmaceutical research is still far from clinical translation, these studies are creative, attractive and have the potential to be beneficial for ICH treatment.

As mentioned above, after ICH onset, lncRNAs dysregulated not only in brain tissue, but also in the circulation system, such as MEG3 and FGD5-AS1 (Hao et al., 2022; Jiang et al., 2022; Wang H. et al., 2022). Also, the association between H19 and the risk of symptomatic ICH in ischemic stroke patients treated with recombinant tissue plasminogen activator was established (Han et al., 2022). The presence of dysregulated lncRNAs in serum, which were also associated with ICH, provided an easier sampling routine and opportunities to use lncRNAs as biomarkers in diagnosing ICH.

On the other hand, the opposite treatments between ischemic stroke and ICH and the nature of rapid pathological changes of both ischemic stroke and ICH require accurate and quick differential diagnosis between these two diseases. With technique development, RNA identification and quantification can be much faster (Carter et al., 2021), which aids the diagnosis and saves time when deciding on proper treatments, especially in rural hospitals (Kamtchum-Tatuene and Jickling, 2019).

Although previous studies demonstrated that mRNAs may have the potential to be biomarkers for the diagnosis and the differentially diagnose of ICH (Stamova et al., 2019), we still lack knowledge about the sensitivity, specificity, and the value of lncRNA for ICH therapy. Opportunities lie ahead, but studies are still needed.

## 4. Conclusion

Previous studies profiled the multi-functional roles of lncRNAs in ICH. lncRNAs are involved in ICH-induced secondary injury, such as inflammatory response, oxidative stress and cell death, etc., via targeting miRNAs or signaling molecules. We are at the infant stage and this field remains attractive. More studies could be conducted to validate the targets of lncRNAs, evaluate the value of lncRNAs as risk prediction for ICH, and develop drugs or special delivery systems to treat ICH by targeting lncRNAs.

## Author contributions

CZ and YZ wrote the manuscript. QW and ZF drew the figure. MZ and XX conducted literature search. TX reviewed and edited the manuscript. All authors contributed to the article and approved the submitted version.

## Funding

This work was supported by the National Key R&D Program of China (2020YFC2008302), the National Natural Science Foundation of China (81673631) and Excellence Development 1·3·5 project of West China Hospital of Sichuan University (ZYJC18028).

## Acknowledgments

This research was supported by National Key Clinical Specialties Construction Program.

## Conflict of interest

The authors declare that the research was conducted in the absence of any commercial or financial relationships that could be construed as a potential conflict of interest.

## Publisher’s note

All claims expressed in this article are solely those of the authors and do not necessarily represent those of their affiliated organizations, or those of the publisher, the editors and the reviewers. Any product that may be evaluated in this article, or claim that may be made by its manufacturer, is not guaranteed or endorsed by the publisher.
