# Supplementary material for: Long non-coding RNAs in intracerebral hemorrhage
Source: Front Mol Neurosci. 2023 Jun 12;16:1119275. doi: 10.3389/fnmol.2023.1119275 (PMC10292654; doi:10.3389/fnmol.2023.1119275)
Supplement: Supplementary file 1 [file Data_Sheet_1.docx]

# PubMed

(("Cerebral Hemorrhage"[Mesh] OR "cerebral hemorrhage" OR "cerebral hemorrhages" OR "intracerebral hemorrhage" OR "intracerebral hemorrhages" OR "ICH" OR encephalorrhagia OR hemorrhagic stroke) OR ("Intracranial Hemorrhages"[Mesh])) AND ("RNA, Long Noncoding"[Mesh] OR "long non-coding RNA" OR "long non coding RNA" OR "long noncoding RNA" OR "long intergenic non-coding RNA" OR "long intergenic non coding RNA" OR "long intergenic noncoding RNA" OR lncRNA)

# WOS

(Cerebral Hemorrhage OR cerebral hemorrhages OR intracerebral hemorrhage OR intracerebral hemorrhages OR ICH OR encephalorrhagia OR hemorrhagic stroke OR Intracranial Hemorrhages) AND (RNA, Long Noncoding OR long non-coding RNA OR long non coding RNA OR long noncoding RNA OR long intergenic non-coding RNA OR long intergenic non coding RNA OR long intergenic noncoding RNA OR lncRNA) (主题)

# MEDLINE (OVIDSP)

1. exp intracranial hemorrhages/

2. exp cerebral hemorrhage/ or exp basal ganglia hemorrhage/ or exp cerebral hemorrhage, traumatic/ or exp cerebral intraventricular hemorrhage/ or exp intracranial hemorrhage, hypertensive/ or exp intracranial hemorrhage, traumatic/ or exp pituitary apoplexy/

3. Cerebral Hemorrhage or cerebral hemorrhages or intracerebral hemorrhage or intracerebral hemorrhages or ICH or encephalorrhagia or hemorrhagic stroke or Intracranial Hemorrhages

4. 1 or 2 or 3

5. exp RNA, Long Noncoding/

6. RNA, Long Noncoding or long non-coding RNA or long non coding RNA or long noncoding RNA or long intergenic non-coding RNA or long intergenic non coding RNA or long intergenic noncoding RNA or lncRNA

7. 5 or 6

8. 4 and 7

# Embase (OVIDSP)

1. exp brain hematoma/ or exp brain hemorrhage/ or exp cerebellum hemorrhage/ or exp massive intracerebral hemorrhage/

2. Cerebral Hemorrhage or cerebral hemorrhages or intracerebral hemorrhage or intracerebral hemorrhages or ICH or encephalorrhagia or hemorrhagic stroke or Intracranial Hemorrhages

3. 1 or 2

4. exp long untranslated rna/

5. RNA, Long Noncoding or long non-coding RNA or long non coding RNA or long noncoding RNA or long intergenic non-coding RNA or long intergenic non coding RNA or long intergenic noncoding RNA or lncRNA

6. 4 or 5

7. 3 and 6

We conducted the search on 2022/10/19 and updated the results before the submission (2022/12/06).
